# Retinoic Acid Supplementation Rescues the Social Deficits in *Fmr1* Knockout Mice

**DOI:** 10.3389/fgene.2022.928393

**Published:** 2022-06-17

**Authors:** Liqin Yang, Zhixiong Xia, Jianhua Feng, Menghuan Zhang, Pu Miao, Yingjie Nie, Xiangyan Zhang, Zijian Hao, Ronggui Hu

**Affiliations:** ^1^ School of Medicine, Guizhou University, Guiyang, China; ^2^ School of Life and Health Sciences, Hangzhou Institute for Advanced Study University of Chinese Academy of Sciences, Hangzhou, China; ^3^ Department of Pediatrics, The Second Affiliated Hospital of Zhejiang University School of Medicine, Hangzhou, China; ^4^ State Key Laboratory of Molecular Biology, Shanghai Institute of Biochemistry and Cell Biology, Center for Excellence in Molecular Cell Science, Chinese Academy of Sciences, Shanghai, China; ^5^ NHC Key Laboratory of Pulmonary Immune-related Diseases, Guizhou Provincial People’s Hospital, Guiyang, China; ^6^ State Key Laboratory of Medical Neurobiology and MOE Frontiers Center for Brain Science, Institute of Science and Technology for Brain-Inspired Intelligence, Fudan University, Shanghai, China

**Keywords:** fragile X syndrome, autism spectrum disorder, retinoic acid, social behavior, FMR1

## Abstract

Autism spectrum disorder (ASD) is a heritable neurodevelopmental disorder with the underlying etiology yet incompletely understood and no cure treatment. Patients of fragile X syndrome (FXS) also manifest symptoms, e.g. deficits in social behaviors, that are core traits with ASD. Several studies demonstrated that a mutual defect in retinoic acid (RA) signaling was observed in FXS and ASD. However, it is still unknown whether RA replenishment could pose a positive effect on autistic-like behaviors in FXS. Herein, we found that RA signaling was indeed down-regulated when the expression of *FMR1* was impaired in SH-SY5Y cells. Furthermore, RA supplementation rescued the atypical social novelty behavior, but failed to alleviate the defects in sociability behavior or hyperactivity, in *Fmr1* knock-out (KO) mouse model. The repetitive behavior and motor coordination appeared to be normal. The RNA sequencing results of the prefrontal cortex in *Fmr1* KO mice indicated that deregulated expression of *Foxp2, Tnfsf10, Lepr* and other neuronal genes was restored to normal after RA treatment. Gene ontology terms of metabolic processes, extracellular matrix organization and behavioral pathways were enriched. Our findings provided a potential therapeutic intervention for social novelty defects in FXS.

## Introduction

Individuals with autism spectrum disorder (ASD) show early-onset social dysfunction and abnormally restricted, repetitive behaviors (Lord et al., 2018). ASD affects approximately one in 44 children, and the incidence is 4-fold higher in males than females (Maenner et al., 2021). The causes of autism are complex, including environmental, genetic and metabolic factors (Peça et al., 2011; Krakowiak et al., 2012; Modabbernia et al., 2017). Fragile X syndrome (FXS) is a X-linked hereditary intellectual disability associated with ASD. FXS mainly results from the abnormal CGG amplification (>200 repeats) of the fragile X mental retardation 1 (*Fmr1*) gene that leads to loss of the expression of fragile X intellectually retarded protein (FMRP) (Saldarriaga et al., 2014). FMRP is an RNA-binding protein that regulates the synaptic development and plasticity (Richter and Zhao, 2021). FXS is the most common genetic cause of ASD, accounting for about 2–6% of the cases (Hogan et al., 2017), and approximately 30% of FXS patients are also diagnosed with ASD (Hagerman and Harris, 2008). Shared symptoms between FXS and ASD, such as repetitive behaviors and social deficits (Kazdoba et al., 2014), indicate an overlap of molecular mechanisms in these diseases (Salcedo-Arellano et al., 2021).

All-trans retinoic acid (RA) is a naturally occurring metabolite from retinol (vitamin A) (Kumar and Duester, 2011). As a critical signaling molecule, RA is involved in synaptic plasticity, neuronal differentiation and brain maturation (Aoto et al., 2008; Chen et al., 2014). Disruption of RA signaling is closely related to the abnormal patterns of the central nervous system, especially the synaptic plasticity homeostasis (Chen et al., 2014). Study in *Fmr1* knock-out (KO) mice, a disease model of FXS, indicated an interaction between FMRP and retinoic acid receptor alpha (RARα), an essential component in RA signaling (Park et al., 2021). Furthermore, RA-mediated synaptic strength regulation was abolished in *Fmr1* KO hippocampal neurons and FXS patient-derived induced pluripotent stem (iPS) cells, thus leading to abnormal synaptic function (Zhang et al., 2018; Zhong et al., 2018). These studies suggested that FXS might result from impaired synaptic plasticity homeostasis caused by dysregulation of RA signaling. Most recently, we have discovered that RA synthesis and RA signaling were down-regulated in the mouse ASD model induced by excessive UBE3A expression, and the ASD-like behaviors caused by repression in RA signaling were successfully ameliorated by oral supplementation of RA in mice (Xu et al., 2018). It was fascinating to ask whether RA replenishment would have beneficial effects on core traits of ASD in *Fmr1* KO mice.

In this study, we have thus first examined whether RA signaling was indeed down-regulated when the expression of *FMR1* was disrupted. Subsequently, we went on to directly test the effect of RA supplementation on the social deficits manifested by the *Fmr1* KO mice, and investigated the potential molecular mechanism by analyzing RNA-seq data. Our findings provide RA replenishment as a potential therapeutic intervention for the social novelty deficit in FXS.

## Materials and Methods

### Animals

*Fmr1* ^(-/y)^ (*Fmr1* KO) mice (aged 2–3 months, FVB background) were gifted from Prof. Chen Zhang of Capital Medical University, Beijing. These mice were then backcrossed for ten generations to our C57BL/6J strain. This *Fmr1* strain was maintained in C57 background. To obtain hemizygous males and WT males, heterozygous females and wildtype males were intercrossed. Mice were housed in a specific-pathogen-free (SPF) facility with 12-h light/dark cycle and *ad libitum* access to food and water. Per cage was housed three to five mice by genotype. All animal experiments were performed strictly in accordance with the instructions of the Institutional Animal Care and Use Committee (IACUC) at the Center for Excellence in Molecular Cell Science, CAS.

### Plasmid Construction

The pGL4-RARE-TK-EGFP-CLPEST plasmid was modified based on pCBG99-Control (Promega) plasmid. The DNA fragment between the two polyA signals, including SV40 promoter, Puromycin and polyA signals, were amplified by PCR from pGL4.22-RARE-TK-luciferase (Xu et al., 2018) plasmid and inserted into pCBG99-Control between XmaⅠ and BamHⅠ. The CL1-PEST sequence from pGL4.22-RARE-TK-luciferase was amplified together with EGFP from pEGFP-N1 plasmid before inserted between XmaⅠ and NheⅠ. The retinoic acid response element (RARE) together with thymidine kinase (TK) promoter were amplified from pGL4.22 and inserted between KpnⅠ and NheⅠ.

The pGL4-RARE-TK-EGFP-CLPEST plasmid contains three copies of DR5 (direct repeat with 5 bp of spacing) variant of RARE in different directions (one in forward direction and the other two in reverse direction according to the sequence) and a EGFP reporter gene with CL1-PEST sequence, which could promote the degradation of EGFP and hence result in rapid turnover of the reporter.

### Cell Line and Transfection

SH-SY5Y(ATCC) cell line was cultured in Dulbecco’s modified Eagle’s medium (DMEM, Corning) supplemented with 10% fetal bovine serum (FBS, Gibco) and 50 μg/ml penicillin/streptomycin (Life Technologies). Cells were maintained at 37°C in a saturated humidity atmosphere containing 5% CO_2_.

SH-SY5Y cells were transfected with indicated plasmids and siRNAs using Lipofectamine 2000 (Thermo Fisher Scientific) according to the manufacturer’s instructions. The siRNA sequences were listed below (Khalil et al., 2008):

si-*FMR1*-1-F: GGG​UGA​GUU​UUA​UGU​GAU​A

si-*FMR1*-1-R: UAU​CAC​AUA​AAA​CUC​ACC​C

si-*FMR1*-2-F: GGA​UGA​UAA​AGG​GUG​AGU​U

si-*FMR1*-2-R: AAC​UCA​CCC​UUU​AUC​AUC​C.

### Flow Cytometry

The cells transfected with siRNAs and plasmids for 48 h were digested and suspended with PBS, before subjected to flow cytometry analysis on Beckman CytoFlex. GFP positive cells were selected and calculated for the proportion. The fluorescence intensity of all live, single cells was also recorded for further analysis.

### RA Administration

Mice (4 weeks old) were administered daily with RA (Sigma, USA) dissolved in olive oil (Aladdin, China) via oral gavage for 1 month, at the dosage of 5 mg/kg. The control group received the olive oil only. Body weights of the mice were measured every 2 days.

### Behavioral Analysis

Male mice at 8 weeks of age were subjected to behavioral tests. Mice were tested at a room with lighting maintained at 230 Lux. Before the experiments began, mice were transferred to the testing room and acclimated for at least 1 h. After each test, wipe the instrument with 75% ethanol to remove any residual odors which may affect subsequent tests. All the behavioral experiments except for self-grooming test and rotarod test were tracked by EthoVision XT (Noldus) tracking system. All data acquisition and analysis were carried out by an individual blinded to the genotype and treatment.

(1) Self-grooming test

The Self-grooming test was performed as previously described (Wang et al., 2020). Mice were placed individually to a clean cage covered with beddings (∼0.5 cm). Prior to the test, animals were allowed to habituate to the novel environment for 10 min. Then the time spent in grooming behaviors was recorded for 10 min. All instances of face-wiping, head and ears scratching/rubbing, and full-body grooming were counted as grooming behavior.

(2) Three-chamber social test

The Three-chamber social test was executed according to previously reported with minor modifications (Rein et al., 2020). In brief, a transparent acrylic box (60 cm × 40 cm × 20 cm) was equally divided into three chambers with removable doors in each partition. Two days prior to the test, the stranger mice (sex and age were matched with test mice) were habituated to the wire cages for 1 h per day. The test mouse was introduced to the central chamber to explore the apparatus freely for 10 min for habituation prior to the experiment.

In the sociability test phase, a stranger mouse (stranger Ⅰ) and an inanimate object were placed into the right and left cages, respectively. The test mouse was allowed to explore all three chambers freely for 10 min and the amount of time spent in each chamber was recorded. Then the test mouse was asked to spend an extra 5 min in the stranger I chamber to get more familiar with stranger I before the next phase.

In the social novelty test phase, the inanimate object was replaced with a novel mouse (stranger II). Similarly, the test animal was allowed to freely explore all three sections of the apparatus for 10 min and the amount of time spent in each chamber was recorded. The sociability preference index = (time spent in stranger I chamber-time spent in object chamber)/(total time in the two chambers); social novelty preference index = (time spent in stranger II chamber-time spent in stranger I chamber)/(total time in the two chambers).

(3) Open-field test

Locomotor activity was evaluated in an acrylic box (40 cm × 40 cm × 40 cm, Med Associates) and videotaped by an overhead camera. The mouse was initially placed in the center of the device and allowed to explore the arena freely for 10 min. The central zone is defined as a 20 cm × 20 cm area in the center of the bottom. The distance travelled and average speed were measured by EthoVision XT (Noldus) tracking system.

(4) Rotarod test

To assess motor coordination and balance, mice were placed on a rotarod apparatus (Columbus Instruments) that accelerates from 4 to 40 rpm for 5 min. The latency to fall was automatically recorded by the infrared detection system. Each mouse was tested for three trials, with 1–2 h between trials in the same day.

### RNA Sequencing

Total RNA samples were extracted from the PFC tissues with Trizol reagent (Tiangen, China) according to the manufacturer’s instruction. PFC tissues from 2 mice of the same genotype and treatment were pooled together as one sample. A total of three samples from six mice in each group were used for high-throughput sequencing. Differential expression was determined using DESeq2. The differentially expressed genes (DEGs) were determined by using 1.5-fold change, with *p* value <0.05 as threshold. GO enrichment analysis of the identified DEGs was performed with ‘clusterProfiler ’package in R. Volcano plots, heatmap and dot plot were drawn in RStudio with the ‘ggplot2’ packages. The generated RNA-seq data have been deposited in the Gene Expression Omnibus (The GEO accession number is: GSE201672).

### Quantitative Real-Time PCR

Total RNA was converted to complementary DNA (cDNA) by using the HiScript^®^ III RT SuperMix for qPCR (+gDNA wiper) (Vazyme, China) according to the manufacturer’s instructions. Quantitative real-time PCR (qRT-PCR) amplifications of various genes were performed using ChamQ universal SYBR qPCR Master Mix (Vazyme, China) in a Roche LightCycler^®^ 384 (Roche, Switzerland). The relative expression level of each transcript was normalized to *Gapdh* using the 2^ΔΔCt^ method. Sequences for the primers used in this study were listed below. All data were obtained from three independent experiments.

**Table:** 

**Gene name**	**Primers**
*GAPDH*	Forward Primer GAG​TCA​ACG​GAT​TTG​GTC​GTA​TTG
	Reverse Primer ATT​TGC​CAT​GGG​TGG​AAT​CAT​ATT​G
*FMR1*	Forward Primer TAT​GCA​GCA​TGT​GAT​GCA​ACT
	Reverse Primer TTG​TGG​CAG​GTT​TGT​TGG​GAT
*Gapdh*	Forward Primer AGG​TCG​GTG​TGA​ACG​GAT​TTG
	Reverse Primer GGG​GTC​GTT​GAT​GGC​AAC​A
*Foxp2*	Forward Primer AGT​GTG​CCC​AAT​GTG​GGA​G
	Reverse Primer CAT​GAT​AGC​CTG​CCT​TAT​GAG​TG
*Xdh*	Forward Primer ATG​ACG​AGG​ACA​ACG​GTA​GAT
	Reverse Primer TCA​TAC​TTG​GAG​ATC​ATC​ACG​GT
*Ccn2*	Forward Primer CCA​ATG​ACA​ATA​CCT​TCT​GC
	Reverse Primer GAA​AGC​TCA​AAC​TTG​ACA​GG
*Arc*	Forward Primer AAG​TGC​CGA​GCT​GAG​ATG​C
	Reverse Primer CGA​CCT​GTG​CAA​CCC​TTT​C
*Lepr*	Forward Primer TGG​TCC​CAG​CAG​CTA​TGG​T
	Reverse Primer ACC​CAG​AGA​AGT​TAG​CAC​TGT
*Serpina3n*	Forward Primer ATT​TGT​CCC​AAT​GTC​TGC​GAA
	Reverse Primer TGG​CTA​TCT​TGG​CTA​TAA​AGG​GG
*Tnfsf10*	Forward Primer ATG​GTG​ATT​TGC​ATA​GTG​CTC​C
	Reverse Primer GCA​AGC​AGG​GTC​TGT​TCA​AGA

### Western Blot

Protein lysates from tissues were extracted using RIPA buffer (50 mM Tris–HCl, 150 mM NaCl, 5 mM EDTA, 0.1% sodium dodecyl sulfate (SDS), 0.5% sodium deoxycholate and 1% NP-40 pH 7.6), supplemented with protease inhibitor cocktail and quantified with a BCA kit (Beyotime, China). The protein lysates were denatured at 100°C for 10 min in 1× SDS loading buffer and then separated by SDS-PAGE. The proteins were transferred to polyvinylidene difluoride membranes (Millipore, Bedford, MA, United States) and blocked in 10% fat-free milk for 1 h at room temperature. Then the membranes were immunoblotted with the primary antibodies overnight at 4°C: anti-FMRP (1:1000, Abcam, ab17722); anti-GAPDH (1:3000, Proteintech, 60004-1-Ig). The corresponding HRP-conjugated secondary antibodies were used at room temperature for 1 h to detect the primary antibody and finally visualized with ECL Western Blotting Reagent (Tanon, Shanghai, China) using Tanon 5200 Imaging System.

### Statistics

Data were analyzed using GraphPad Prism 8.0 software (GraphPad Software, San Diego, CA, United States). Statistical tests were conducted as stated in the figure legends. Values are presented as means ± SEM.

## Results

**FIGURE 1:**
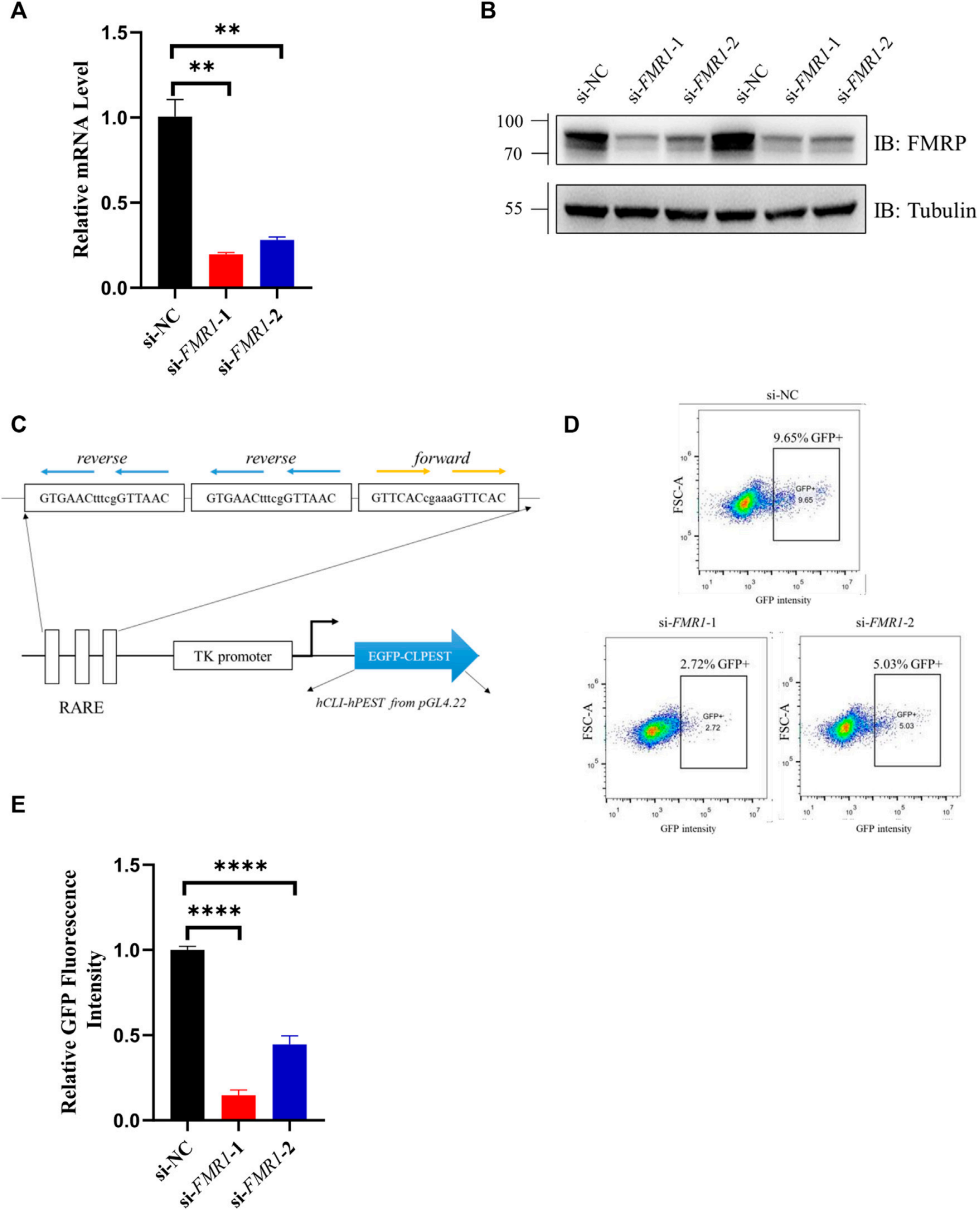
RA-induced gene expression was down-regulated in *FMR1* knockdown cells. **(A,B)** Analysis with **(A)** RT-qPCR and **(B)** Western blotting for the effects of siRNAs on the expression level of *FMR1* in SH-SY5Y cells. **(C)** Schematic diagram of 3×RARE-EGFP reporter construct. The expression of EGFP was regulated by three copies of RA-response element (RARE). CL1PEST sequence was attached to the C terminal of EGFP to promote its turnover. **(D)** Flow cytometry analysis of SH-SY5Y cells transfected with RARE-EGFP reporter and indicated siRNAs. The proportion of GFP positive (GFP+) cells were noted **(E)**. Quantification of normalized GFP fluorescence intensity from flow cytometry analysis. n = 3 biological replicates. Data are presented as means ± SEM. **p < 0.01, ****p < 0.0001. One-way ANOVA with Dunnett *post hoc* test.

To investigate whether RA signaling was down-regulated when the expression of *FMR1* was decreased, we first knocked down *FMR1* gene expression by siRNA in SH-SY5Y cells, a commonly used cell line in RA research (Cheung et al., 2009). The mRNA level (Figure 1A) and protein level (Figure 1B) of *FMR1* were markedly decreased in cells transfected with siRNAs targeting *FMR1*. We then co-transfected SH-SY5Y cells with siRNA and a GFP reporter, the expression of which was driven by RA-response element (RARE), to examine the RA signaling (Figure 1C). The proportion of GFP positive cells (Figure 1D) and the mean value of GFP fluorescence intensity (Figure 1E) were decreased in cells with *FMR1* siRNAs. Overall, these results suggested that RA signaling was indeed down-regulated in cells with decreased level of *FMR1*.

**FIGURE 2:**
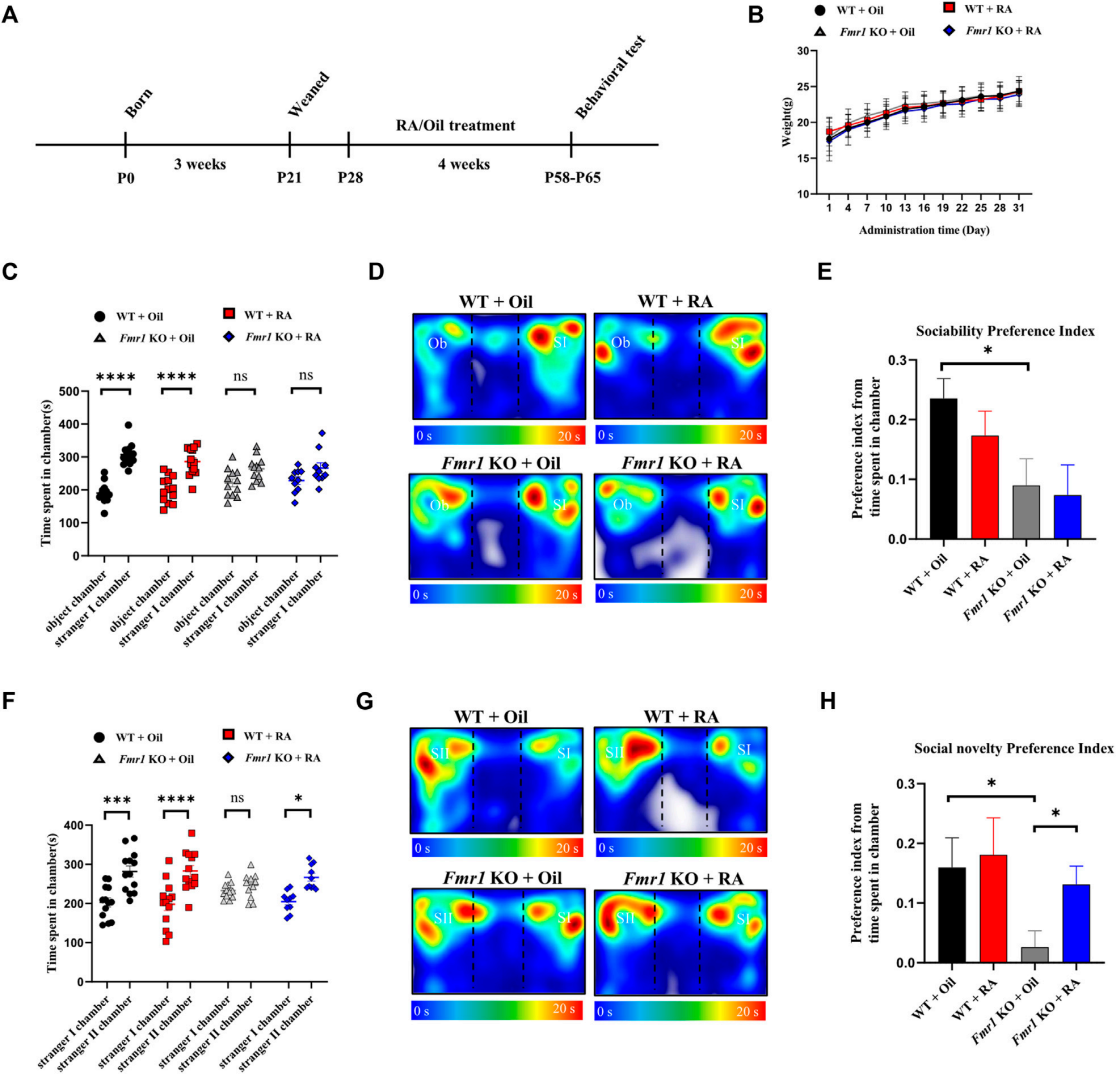
RA administration restores social novelty behavior in *Fmr1* KO mice. **(A)** Schematic diagram of experimental design. WT and *Fmr1* KO male mice were orally administered with Oil or RA (5 mg/kg/d) for 4 weeks, before subjected to behavioral tests at postnatal day P58-P65. **(B)** Body weights of mice recorded every 4 days for 1 month after Oil or RA administration. WT + Oil (n = 13), WT + RA (n = 13), *Fmr1* KO + Oil (n = 13), *Fmr1* KO + RA (n = 12). **(C–E)** Time spent in each chamber **(C)**, representative heat map **(D)** and the preference index **(E)** for the sociability test performed with 4 groups of mice. The Ob and SI indicate the object and stranger Ⅰ, respectively. WT + Oil (n = 13), WT + RA (n = 13), *Fmr1* KO + Oil (n = 12), *Fmr1* KO + RA (n = 10). **(F–H)** Time spent in each chamber **(F)**, representative heat map **(G)** and the preference index **(H)** for the social novelty test performed with 4 groups of mice. The SI and SII indicate the stranger Ⅰ and stranger Ⅱ, respectively. WT + Oil (n = 13), WT + RA (n = 13), *Fmr1* KO + Oil (n = 12), *Fmr1* KO + RA (n = 10). Data are presented as means ± SEM. *p < 0.05, ***p < 0.001, ****p < 0.0001, ns, not significant. **(B,C,F)** Two-way ANOVA with Bonferroni *post hoc test*; **(E,H)** Unpaired two-tailed *t* test with Welch’s correction.

We used male *Fmr1* KO mice (*Fmr1* ^(-/y)^) and their wild-type (WT) littermates to explore the effect of RA exerting on the behaviors. Both WT and KO mice were treated with RA by oral gavage at 5 mg/kg/day or olive oil as control from 4 weeks of age for 1 month as previously described (Pasqualetti et al., 2001; Xu et al., 2018), followed by behavior tests at postnatal day 58 (Figure 2A). During the intragastric administration, no significant weight differences were observed among the four groups of mice (WT + Oil, WT + RA, KO + Oil, KO + RA) (Figure 2B). The shared symptoms between FXS and ASD are impaired social skills and repetitive, stereotyped behaviors (Kazdoba et al., 2014), which were tested by the Three-chamber social task and Self-grooming task. Compared with WT mice, *Fmr1* KO mice spent comparable time with an object or a live mouse regardless of RA administration (Figures 2C,D), manifesting impaired sociability (Figure 2E). WT mice spent longer time with a novel mouse (stranger II) than with a familiar mouse (stranger I), while *Fmr1* KO mice, if not treated with RA, spent similar time in each chamber (Figures 2F,G), showing defects in social novelty behavior (Figure 2H). The supplementation of RA, however, significantly increased the time that KO mice spent with a novel stranger, thus restoring the defective social novelty behavior (Figures 2F,H). We found that RA supplementation could rescue the deficits in social novelty, yet not in sociability, of *Fmr1* KO mice, without significantly changing the behaviors of the WT mice.

**FIGURE 3:**
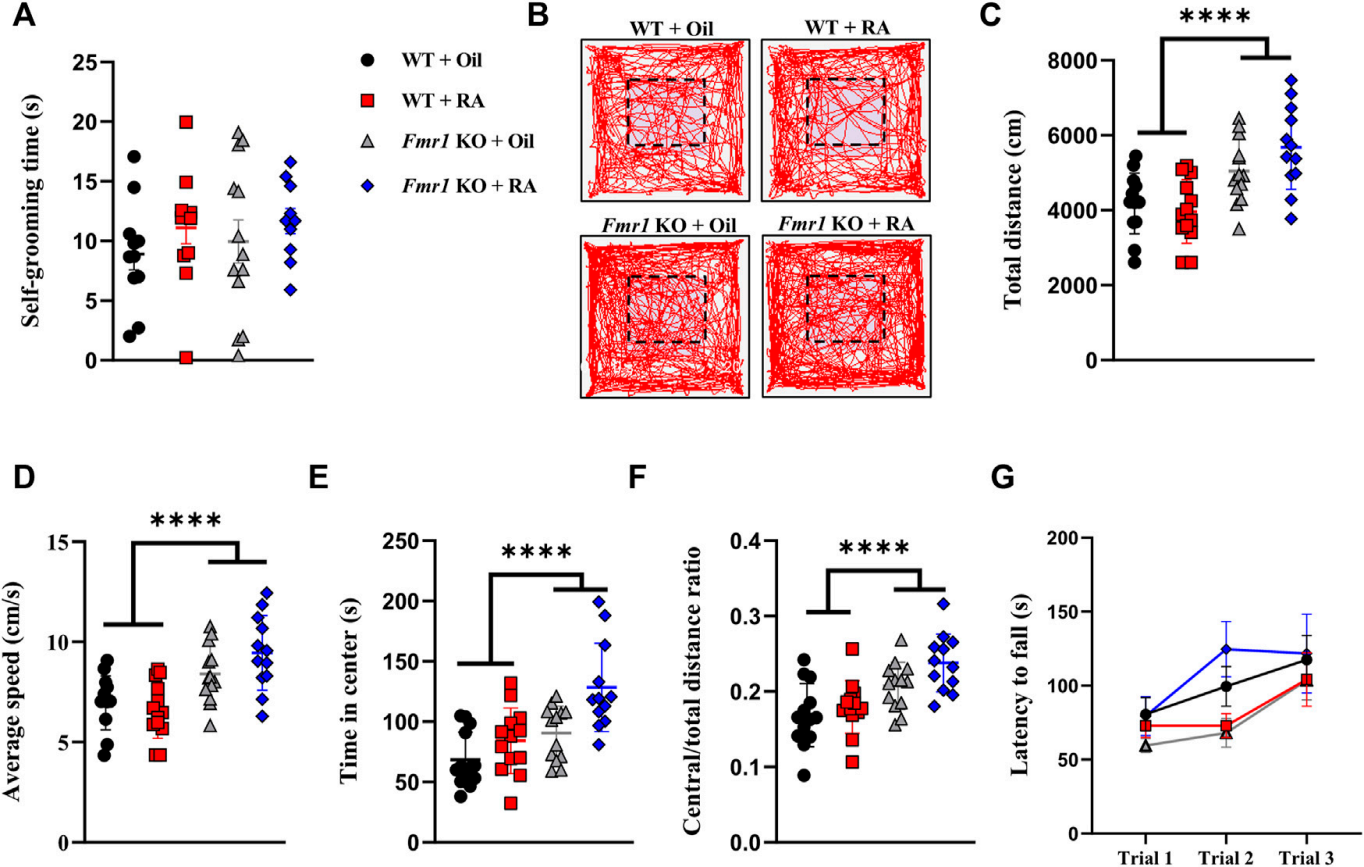
RA administration does not affect repetitive behavior, locomotion, or motor coordination in *Fmr1* KO mice. **(A)** Time spent in self-grooming for repetitive behavioral test performed with 4 groups of mice. WT + Oil (n = 11), WT + RA (n = 12), *Fmr1* KO + Oil (n = 13), *Fmr1* KO + RA (n = 10). **(B–F)** Representative activity traces **(B)**, total distance moved **(C)**, average speed **(D)**, time spent in center **(E)**, and ratio of central distance to total distance **(F)** in the open-field test performed with 4 groups of mice. The dotted line indicates the central area. WT + Oil (n = 13), WT + RA (n = 13), *Fmr1* KO + Oil (n = 13), *Fmr1* KO + RA (n = 12). **(G)** Latency to falling in the rotarod test performed with 4 groups of mice. WT + Oil (n = 11), WT + RA (n = 12), *Fmr1* KO + Oil (n = 13), *Fmr1* KO + RA (n = 11). Data are presented as means ± SEM. ****p < 0.0001. **(A)** One-way ANOVA with Bonferroni *post hoc test*; **(C–G)** Two-way ANOVA with Bonferroni *post hoc* test.

We then examined the repetitive behavior, the other core symptom of ASD, in *Fmr1* KO mice. We found no significant difference in self-grooming time, a manifestation of repetitiveness, either with or without RA treatment (Figure 3A). In addition to the behavioral study of mutual symptoms mentioned above, motor activity and coordination in *Fmr1* KO mice were also detected. Consistent with previous studies (Ding et al., 2014; Gantois et al., 2017; Nolan et al., 2017), KO mice showed increased travel distance and average speed in the open field test, indicating hyperactivity, which was not ameliorated by RA (Figures 3B–D). The time spent in central area (Figure 3E) and the ratio of total distance travelled in the central area (Figure 3F) were significantly increased in *Fmr1* KO mice, suggesting that *Fmr1* KO mice manifested reduced anxiety-like behavior compared with the WT mice, which was consistent with other reports (Yan et al., 2004; Zieba et al., 2019). The motor coordination was not significantly affected by either elimination of *Fmr1* expression or RA treatment (Figure 3G). Taken together, these results suggest that RA supplementation can alleviate the defects in social novelty, but not in sociability or hyperactivity, in *Fmr1* KO mice.

**FIGURE 4:**
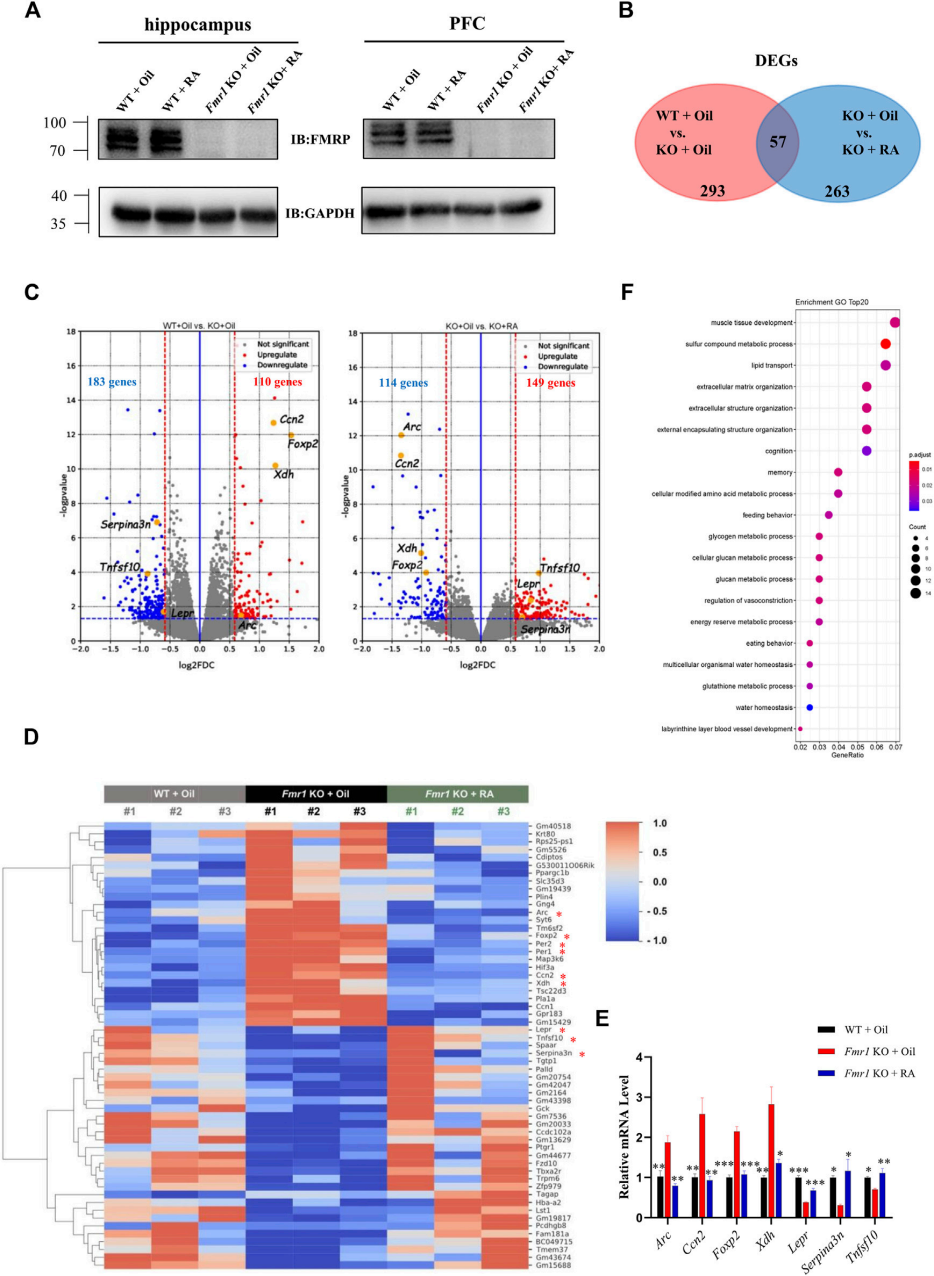
RA administration restores mRNA expression in the PFC of *Fmr1* KO mice. **(A)** Representative western blots of FMRP proteins in the hippocampus and prefrontal cortex from 4 groups of mice. (n = 3 per group). **(B)** Venn diagram for overlap analysis (57 genes) between differentially expressed genes (DEGs) in WT + Oil vs KO + Oil groups (293 genes) and DEGs in KO + Oil vs KO + RA groups (263 genes). **(C)** Volcano plots for differentially expressed genes (DEGs). Left: WT + Oil and KO + Oil groups; Right: KO + Oil and KO + RA groups. Blue dots represent down-regulated genes while red dots represent upregulated genes. The blue dashed line indicates *p* = 0.05. The red dashed lines indicate ∣FoldChange∣> 1.5 (∣log2(FoldChange)∣> 0.5849). (n = 3 pooled from six mice per group). **(D)** Heatmap represents the changes in expression of overlapped genes (57 genes). Blue stripes indicate low expression level; red stripes indicate high expression level. Genes mentioned in the main texts were marked with an asterisk. **(E)** Quantitative real-time PCR analysis of indicated genes mRNA expression in the prefrontal cortex of mice from WT + Oil, KO + Oil and KO + RA groups. (n = 3 samples pooled from six mice. **(F)** Top 20 biological process (BP) pathways in the Gene Ontology (GO) enrichment analysis. Data are presented as means ± SEM. *p < 0.05, **p < 0.01, ***p < 0.001. **(E)** One-way ANOVA with Bonferroni *post hoc* test.

The behavioral results have revealed the therapeutic potential of RA for rescuing aberrant social novelty behavior. Since RA treatment did not restore the protein level of FMRP in the hippocampus or prefrontal cortex (PFC) of *Fmr1* KO mice (Figure 4A), we performed RNA sequencing (RNA-seq) of PFC samples from three groups of mice (WT + Oil, KO + Oil, KO + RA) to acquire further insight into the underlying mechanisms of RA treatment. The PFC region has been shown to be one of the primary brain regions that regulating social behaviors (Amodio and Frith, 2006; Brumback et al., 2018). Differentially expressed genes (DEGs) (∣FoldChange∣> 1.5, *p* value <0.05) were identified by comparing the sequencing results between WT + Oil and KO + Oil, as well as KO + Oil and KO + RA. As shown in the Venn diagram (Figure 4B), 293 and 263 DEGs were found, respectively, with 57 of them overlapped (Detailed information in Supplementary Table S1). In specific, compared with WT + Oil group, 110 genes were up-regulated and 183 genes were down-regulated in KO + Oil group. While compared with KO + Oil group, there were 149 up-regulated genes and 114 down-regulated genes in KO + RA group (Figure 4C). Our intention was to find out the DEGs in the KO + Oil group, of which the expression levels were restored to the similar level as those in the WT + Oil group after RA supplementation. As shown in Figures 4D, 56 out of 57 overlapped DEGs (except *Tagap*, T cell activation RhoGTPase activating protein) meet the criteria mentioned above, including some autism-related genes (Simons Foundation Autism Research Initiative, SFARI), such as period circadian clock 1 (*Per1*), period circadian clock 2 (*Per2*) and forkhead box P2(*Foxp2*). Several DEGs associated with neuronal functions were also identified (Figure 4C). The abnormal increase in the expression of activity-regulated cytoskeleton-associated protein (*Arc*), cellular communication network factor 2 (*Ccn2*), forkhead box P2 (*Foxp2*), and xanthine dehydrogenase (*Xdh*) in *Fmr1* KO mice was down-regulated after RA administration, while the expression level of leptin receptor (*Lepr*), serine peptidase inhibitor clade A member 3N (*Serpina3n*) and tumor necrosis factor superfamily member 10 (*Tnfsf10*) was increased to normal as WT. These findings were verified by quantitative Real-time PCR (qRT-PCR) (Figure 4E). In order to probe the functional associations of DEGs caused by RA supplementation, we performed Gene Ontology (GO) enrichment analysis on the DEGs between KO + Oil and KO + RA groups, and identified significant changes in 40 terms of Biological Process (BP) (*P*, adjust <0.05, complete list in Supplementary Table S2; top 20 pathways in Figure 4F). The most enriched pathways were various metabolic processes, such as glycogen metabolic, glucan metabolic and glutathione metabolic, etc. Besides, the pathways associated with memory, cognition, eating behavior and extracellular matrix organization were also enriched. The alterations of these pathways were previously implicated in FXS (Lumaban and Nelson, 2015; O’Leary and Nolan, 2015; Reinhard et al., 2015; Bostrom et al., 2016; Westmark, 2021). Collectively, these results suggested that RA alleviated defective social novelty behavior in *Fmr1* KO mice possibly through restoring anomalous expressed genes and biological processes to normal.

## Discussion

*Fmr1* is a strong candidate gene associated with ASD, and its deficiency was implicated in autism development (Niu et al., 2017). Several potential treatments have been proved to be able to alleviate abnormalities in *Fmr1* KO mice through different pathways. Application of dopamine rescued the impaired social novelty behaviors by reduction of striatal tyrosine hydroxylase protein (Chao et al., 2020). Weekly treatment with purinergic antagonist suramin restored the social behaviors by regulating purinergic signaling (Naviaux et al., 2015). Metformin, a widely-used anti-diabetic drug, was found to rescue the social novelty deficit, repetitive behaviors, and abnormal incidence of seizures in *Fmr1* KO mice through normalizing ERK signaling (Gantois et al., 2017). Recently, increasing amount of evidence has suggested an association between impaired RA signaling and ASD (Pavăl et al., 2017; Chen et al., 2018; Zhou and Li, 2018; Hao et al., 2019). Furthermore, the perturbation of synaptic plasticity homeostasis mediated by RA was observed in *Fmr1* KO mice and FXS patient-derived induced pluripotent stem (iPS) cells (Soden and Chen, 2010; Zhang et al., 2018). This prompted us to investigate the role that RA plays in FXS behavioral traits.

Our work demonstrated that *Fmr1* KO mice displayed atypical social behaviors and hyperactivity, yet no defect in repetitive behavior or motor coordination was noted. RA replenishment rescued social novelty behavior, probably due to the normalization of anomalous gene expression and defective pathways. Since the synaptic plasticity homeostasis mediated by RA was abolished in *Fmr1* KO neuron (Soden and Chen, 2010; Sarti et al., 2013), the treatment with RA could not increase the mEPSC amplitude as in WT neurons. This suggested that the improvement in social novelty behavior induced by RA might not result from the restoration in synaptic strength, but from a transcriptional regulation of neuronal genes. Therefore, we performed RNA-seq and identified several genes associated with behavioral traits.

Specifically, the mRNA level of *Ccn2*, the connective tissue growth factor that negatively regulates myelination (Ercan et al., 2017), was restored to normal after RA administration. *Foxp2*, a transcription suppressor related to the social defects in ASD patients (Chien et al., 2017), was found irregularly increased in *Fmr1* KO mice. Its excessive expression could result in transcription inhibition of mesenchymal-epithelial transition factor (*MET*) and lead to abnormal neuronal differentiation and growth (Mukamel et al., 2011). The mRNA level of *Lepr*, whose insufficient level could cause impaired social interaction (Meyer et al., 2014), was decreased when the *Fmr1* gene was knocked out. *Tnfsf10*, also known as tumor necrosis factor-related apoptosis-inducing ligand (*TRAIL*), encodes a membrane-bound cytokine that induces cellular apoptosis (Park et al., 2015). Research implied a contribution from defective programed cell death to the excessive synaptic connections in *Fmr1* mutants and behavioral phenotype of children with FXS (Gatto and Broadie, 2011; Cheng et al., 2013), which was also in accordance with our finding that *Tnfsf10* mRNA level was significantly decreased in *Fmr1* KO mice. RA supplementation restored the expression of these genes (Figure 4E) and normalized neuronal function, which might ameliorate social behaviors in the end.

The biological process pathways enriched in the GO analysis were also found related to the FXS. For instance, extracellular structure plays a pivotal role in neurite outgrowth, neural connectivity, and synaptic plasticity (Cope and Gould, 2019; Peteri et al., 2021). Alterations in connected tissue and extracellular matrix (ECM) have been implied in the pathophysiological development of FXS (Ramírez-Cheyne et al., 2019). According to our GO analysis result, three GO terms concerning extracellular structure were enriched within TOP six terms. This suggested that RA treatment might significantly improve the neural connectivity in the altered ECM from the PFC in *Fmr1* KO mice.

The enrichment in glycogen and glucan metabolic process pathways induced by RA (Figure 4F) brought our attention to glycogen synthase kinase 3 (GSK3), the inhibition of which was proved to improve the impaired behaviors of ASD and FXS (Franklin et al., 2014; McCamphill et al., 2020; Rizk et al., 2021). Some reports demonstrated that inhibition of GSK3 could enhance retinoic acid receptor activity (Si et al., 2011). These researches indicated a potential link between enhanced RA signaling and restored symptoms in FXS. It is worth mentioning that knockout of *Fmr1* or supplementation of RA did not significantly change the mRNA level of *Gsk3a* or *Gsk3b* (data not shown). A crosstalk with RA signaling and GSK3 activity might exist.

The impairment of cognitive abilities and infant diet, was previously implicated in the individuals with FXS (Bostrom et al., 2016; Westmark, 2021). The corresponding GO terms of them, cognition and eating behaviors, were also enriched after RA administration (Figure 4F).

RA has been used for the treatment of several diseases, like acute myelocytic leukemia (Stahl and Tallman, 2019) and skin disorders (Szymański et al., 2020), which suggests the safety of RA and its potential to be used for other diseases. The challenge here is that RA has poor solubility in aqueous solutions, so it is rather difficult to reach an effective concentration in tissues, like brain (Ferreira et al., 2020). In order to increase the stability of RA in human body and the selectivity against RARs, synthetic retinoids have been developed for clinical trials of neurological diseases, for example, Alzheimer’s disease (Wołoszynowska-Fraser et al., 2020). These studies shed light on the possibility of RA treatment in FXS and ASD patients in the future.

Although many questions remained to be addressed, our findings that RA supplementation improved social novelty behavior in *Fmr1* KO mice provided a potential therapeutic intervention for FXS, which may further be used in other disease models with defective RA signaling.

## Data Availability

The datasets presented in this study can be found in online repositories. The names of the repository/repositories and accession number(s) can be found in the article/supplementary material.
